# Co-creating and Mapping an Exclusive Breastfeeding Framework among Latino Populations in the United States: An Integrated Framework Adaptation Process and Scoping Review

**DOI:** 10.1016/j.advnut.2025.100483

**Published:** 2025-07-19

**Authors:** Armando Peña, Zoe Barnsfather, Alison M Miller, Ashley Alvarado, Deanna Reinoso, Melissa Klitzman, Ann Marie Neeley, Ana Maria Linares, Katherine Harkov, Tess Phillips, Amanda Santiago, Christine Spencer, Fernanda Betti, Julie A Patterson, Ines Casanova, Karla Baquerizo, Kiran Snow, Angelica Maria Mays, Shannon Lopez, Courtnie Leeper, Rose Douglass, Emily Lynch, Freedom Kolb, Erin M Cleary, Kundai Crites, Amy Minix, Richard J Holden

**Affiliations:** 1 Department of Health and Wellness Design, School of Public Health, Indiana University, Bloomington, IN, United States; 2 College of Liberal Arts and Sciences, Indiana University, Bloomington, IN, United States; 3 School of Medicine, Indiana University, Indianapolis, IN, United States; 4 Regenstrief Center for Health Equity Research, Eskenazi Health, Indianapolis, IN, United States; 5 Division of Neonatal Perinatal Medicine, Department of Pediatrics, Riley Hospital for Children at the Maternity Tower NICU, Indianapolis, IN, United States; 6 Division of Nutrition and Physical Activity, Indiana Department of Health, Indianapolis, IN, United States; 7 College of Nursing, University of Kentucky, Lexington, KY, United States; 8 Nurse-Family Partnership-Goodwill Southern and Central Indiana, Indianapolis, IN, United States; 9 Supplemental Nutrition Assistance Program for Women, Infants, and Children-Marion County, Indianapolis, IN, United States; 10 Marion County Public Health Department, Marion County, IN, United States; 11 Riley Maternity and Newborn Health, Indianapolis, IN, United States; 12 Elige Amamantar, Indianapolis, IN, United States; 13 Ascension St. Vincent Women’s Hospital, Indianapolis, IN, United States; 14 School of Health Studies, Northern Illinois University, DeKalb, IL, United States; 15 Department of Applied Health Sciences, School of Public Health-Bloomington, Indiana University, Bloomington, IN, United States; 16 Self-representation, Indianapolis, IN, United States; 17 Department of Perinatal-Neonatal Medicine, Indiana University, Indianapolis, IN, United States; 18 Pre to 3, Vanderburgh County Health Department, Vanderburgh, IN, United States; 19 Eskenazi Health, Indianapolis, IN, United States; 20 Maternal and Child Health Division, Indianapolis, IN, United States; 21 WIC Division, Indiana Department of Health, Indianapolis, IN, United States; 22 The Milk Bank, Indianapolis, IN, United States; 23 Department of Obstetrics and Gynecology, Indiana University, Indianapolis, IN, United States; 24 Division of Maternal-Fetal Medicine, Department of Obstetrics and Gynecology, School of Medicine, Indiana University, Indianapolis, IN, United States; 25 Sciences Library, Indiana University, Bloomington, IN, United States

**Keywords:** noncommunicable disease, prevention, diabetes, nutrition, neonate, early life, obesity, health equity, health disparities

## Abstract

Increasing exclusive breastfeeding among Latino populations has the potential to reduce health disparities. There is a need for a multilevel and multidomain framework of exclusive breastfeeding determinants. This study aimed to co-create an exclusive breastfeeding determinants framework among Latino populations and map this framework using the current literature. Our community coalition convened in working groups to adapt a multilevel and multidomain determinants framework with 20 cells (4 levels × 5 domains) for exclusive breastfeeding among Latino populations. We documented all referenced determinants in working groups, and 2 independent raters deductively and inductively analyzed these specific determinants into themes by cell (level domain). An integrated scoping review mapped the determinants addressed in the literature of exclusive breastfeeding interventions among Latinos in the United States onto the framework cells. Two independent raters transcribed intervention descriptions verbatim and deductively analyzed the text using our list of determinants as the codebook. Inductive analysis allowed for emerging determinants. We mapped determinants that were addressed by theme. A total of 111 specific determinants were referenced in working groups that were categorized into 53 determinant themes. Most studies addressed Individual-level determinants at each domain (*n* = 11–16 studies) except for Built Environment (*n* = 3). At the Interpersonal level, Behavior (*n* = 11) and Health Care System (*n =* 16) domains were predominantly addressed. At the Community level, Built Environment (*n =* 14) and Health Care System (*n =* 15) domains were addressed. Most studies at the Societal level addressed the Health Care System domain but none addressed Biological, Behavior, or Built Environment domains. Extension of care, culturally relevant care, knowledge and skills, mother–infant bonding, and practitioner–dyad relationship were referenced the most of all 56 themes (*n ≥* 13 each). Increasing exclusive breastfeeding among Latinos is a multifaceted challenge. Innovative areas for future work include Biological and Sociocultural domains beyond the Individual level as well as most domains at the Societal level.

**Statement of significance**

*La Cosa Más Buena* provides a multilevel and multidomain framework for others in the field to adopt and adapt according to specific local contexts among vulnerable populations. The mapping of the framework identified key areas that are ripe for future innovations.

## Introduction

Exclusive breastfeeding is defined by the WHO as feeding an infant only human milk, and nothing else [1], and is recommended for the first 6 mo of life by national and global health organizations [[2], [3], [4]]. Exclusive breastfeeding, compared with formula feeding, has been associated with a reduced risk of noncommunicable diseases that disproportionately burden Latino children (e.g., obesity, type 2 diabetes) and mothers (e.g., type 2 diabetes, breast cancer, ovarian cancer) compared with non-Hispanic White populations [[5], [6], [7], [8], [9], [10], [11]]. However, Latino infants in the United States are exclusively breastfed at suboptimal rates (25.9%) [12]. As a reference, the national Healthy People 2030 goal for exclusive breastfeeding at 6 mo is 42.4% for 2030 [13,14]. Achieving or exceeding the new 2030 goal for exclusive breastfeeding among Latinos has the potential to reduce health disparities, but it is a grand challenge considering that it will require a doubling effect from current rates [14,15].

Paradoxically, Latinos have demonstrated the highest rates of intentions to breastfeed and ever breastfeeding in the first year of life, compared with other racial and ethnic populations, including non-Hispanic White [13,16]. Despite high breastfeeding rates, the exclusivity of human milk is interrupted by high rates of early introduction of formula within the first month of life [17]. This mixed feeding approach of breastfeeding and formula feeding is a norm in Latino culture referred to as *las dos cosas* (“both things”). In addition to cultural norms, many other determinants of exclusive breastfeeding have been described among Latino populations that operate beyond the Individual level and across multiple life course domains, including high-quality lactation care [18], unmet social needs like food insecurity and financial strain [19], increased obesity and diabetes prevalence [20], breastfeeding policies and initiatives, and regulation of commercial milk formula marketing [21,22]. Hence, interventions that target increases in exclusive breastfeeding warrant multifaceted approaches.

The National Institute on Minority Health and Health Disparities (NIMHD) Research Framework is a determinants framework that integrates a socioecological model with a life course model [23]. The framework was disseminated “for use… in its original form or adapted to apply to a particular research question or population. The framework is also intended to be used as a tool to assess the state of current research and identify gaps and opportunities for future research.” To guide our local efforts in Indiana, our team established and engaged a community coalition, *Lactancia para Los Hoosiers* (Lactation for Latino Hoosiers), to adapt the NIMHD framework for increasing exclusive breastfeeding among Latino populations. We also mapped the newly created framework in a scoping review based on determinants that have been addressed in the literature to identify ripe areas for future innovations [24,25].

## Methods

### Study design

Our study integrates qualitative and quantitative components in 2 phases: *1*) framework adaptation and *2*) framework mapping. In the first phase, our coalition collaborated in working groups to adapt the NIMHD Research Framework. In the second phase, we conducted a scoping review to map the adapted framework using the current literature of exclusive breastfeeding interventions among Latino populations.

### Framework adaptation

#### Coalition

The coalition, *Lactancia para Los Hoosiers* (translated as “Lactation for Latino Hoosiers”), included a diverse group of 22 community partners who are invested in enhancing lactation care among Latino populations to increase exclusive breastfeeding. For reference, a Hoosier refers to a resident or native of Indiana. The team included position titles of International Breastfeeding Certified Lactation Consultants (IBCLC; *N =* 8), academic researchers (*N =* 5), breastfeeding coordinators at federal programs (*N =* 3), obstetricians and gynecologists or maternal-fetal medicine doctors (*N =* 3), pediatricians (*N =* 2), registered dietitians (*N =* 2), breastfeeding peer counselors (*N =* 1), a doula (*N =* 1), the statewide breastfeeding initiatives manager (*N =* 1), an Social Determinants of Health (SDOH) medical director of the third largest safety net health system in the United States (*N =* 1), a registered nurse (*N =* 1), and a community lactation advocate with lived breastfeeding experience in the United States as a Latina immigrant (*N =* 1) (total *N* of individuals in coalition is >22 because of overlapping professions). Entities represented included academic institutions, a large safety net health system, the local children’s hospital, a community women’s hospital, the state health department, lactation and parent care and home visiting programs, the local milk bank, a privately Latina-owned lactation care business, and the local community. The coalition was established purposefully so that the data collected would reflect diverse perspectives on the contexts of exclusive breastfeeding among the local Latino Hoosier community.

#### Working groups

Each coalition member, based on their interests, opted in ≤5 separate working group sessions that correspond to the 5 life course domains of the NIMHD framework. Sessions were held using videoconference technologies (Zoom Video Communications, Inc.) and for 4 h at a time to accommodate the schedules of coalition members. Working groups were structured into 2 4-h sessions in 1 d for each domain. Members were advised to join the sessions corresponding to their subscribed domains for as long as they desired and were able. A moderator (AP) guided discussion focused on the domain of each respective session and documented all referenced determinants onto a spreadsheet that was visible to the group. When referencing determinants, working groups were asked to use their own work and lived experiences to consider key determinants of exclusive breastfeeding among Latino populations. These determinants were recorded as terms or phrases in a Microsoft Excel spreadsheet for further analysis. Unstructured notes were also taken throughout the working groups.

#### Analysis

Two independent raters (AP and ZB) reviewed all determinants referenced in the spreadsheet to, first, determine whether the determinant was relevant to Latino populations. For any terms or phrases that were unclear upon review, the raters referred to unstructured notes or followed up with coalition members for clarification. Once the spreadsheet was finalized, the 2 independent raters assigned each specific determinant to the corresponding level(s) of influence of the framework. For example, the phrase “Paternity leave policies” would be referenced as a Societal-level determinant. Raters used the original NIMHD Research Framework determinants as a codebook in a deductive analysis to generate determinant themes but also allowed for emerging determinants (inductive analysis). The 2 raters convened to build consensus on the finalized represented levels of influence and determinant themes that were derived from the specific determinants. Any disagreements on either of these 2 components (assigning levels, thematic analysis) between the 2 raters were resolved with input from a third rater.

#### Adaptation

Using the original template of the NIMHD Research Framework, we filled in each cell according to respective determinant themes. Last, we worked with a graphics designer to develop a graphic that appropriately reflected the newly created framework that could be used in future dissemination efforts.

### Framework mapping

#### Scoping review

The PRISMA for Scoping Reviews was used to map the current literature among Latinos (Population) in interventions that targeted exclusive breastfeeding (Concept) to identify determinants of exclusive breastfeeding that have been addressed (Context) [26]. This PCC mnemonic (Population, Concept, Context) “is recommended as a guide to construct a clear and meaningful title and inclusion criteria for a scoping review” [24] and is described at the preregistration site at Open Science Framework (https://osf.io/e635z/) [27].

#### Eligibility criteria

Studies were included in the analysis if they were intervention studies that measured exclusive breastfeeding in response to the interventions and enrolled predominantly Latino study samples (≥50% Latino). Given the focus on exclusive breastfeeding disparities among Latinos in the United States, studies conducted outside the United States were excluded. Any interventions conducted among high-risk infants admitted to the neonatal intensive care unit were excluded. All studies in the literature were considered with no limitation on publication date.

#### Data sources and search strategy

The electronic databases of PubMed, Web of Science, and Embase were searched for potentially eligible articles and the search terms for each database are provided in the supplementary material and also publicly available at the preregistration site [27]. No constraints were placed on the dates of publication of studies. The search for potentially eligible articles occurred between March 2024 and April 2024.

#### Study selection

Articles were uploaded into Covidence, an online scoping review platform for screening. Duplicates were identified electronically, confirmed manually, and removed. The remaining titles and abstracts of all studies were screened by a primary and secondary reviewer to consider including in the full-text review process. Full-text reviews were conducted by the primary and secondary reviewers to identify studies that met inclusion criteria. Disagreements between primary and secondary reviewers warranted the inclusion of a third reviewer for consensus on a given article’s eligibility.

#### Data extraction, synthesis, and analysis

A data extraction spreadsheet in Microsoft Excel was used for the collection of study characteristics (e.g., first author, year of publication, geographical location, single or multisite, sample size, etc.), sample characteristics, components of interventions, and characteristics of infant feeding components.

To assess exclusive breastfeeding determinants that were noted in the adaptation phase and addressed in other studies, we transcribed the descriptions of all interventions from original articles, protocol papers, or preregistration sites verbatim into a Microsoft Word document. Two independent raters (AS and AMM) conducted a deductive analysis using the predetermined thematic categories from the newly adapted framework to guide the mapping of other intervention studies. Inductive analysis was also used to allow for emerging determinant themes. On completion of mapping determinants themes, the 2 raters convened to reach consensus on the finalized list of themes. Any disagreements warranted inclusion of a third reviewer. We tallied the numbers of domains and levels that were addressed across all studies as a count and percentage. We also tallied the number of studies that addressed each framework cell (e.g., Individual-Health Care System).

## Results

### Adaptation phase results

**FIGURE 1:**
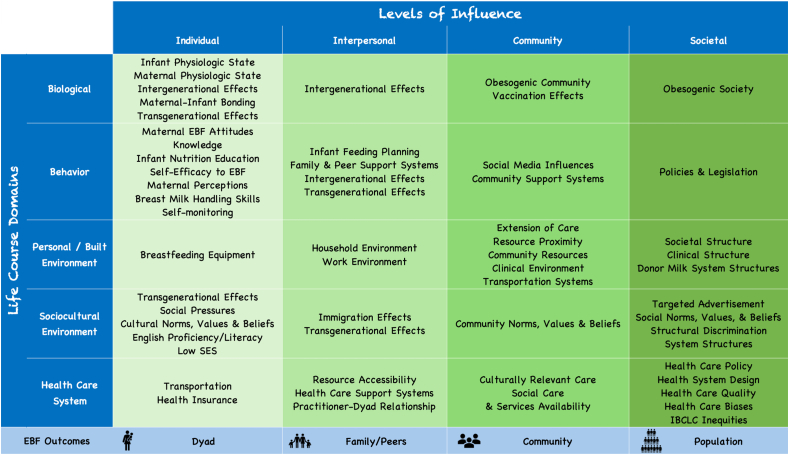
Adapted NIMHD Research Framework—exclusive breastfeeding in Latinos. EBF, exclusive breastfeeding; IBCLC, International Board Certified Lactation Consultant; SES, socioeconomic status; NIMHD, National Institute of Minority Health and Health Disparities.

**FIGURE 2:**
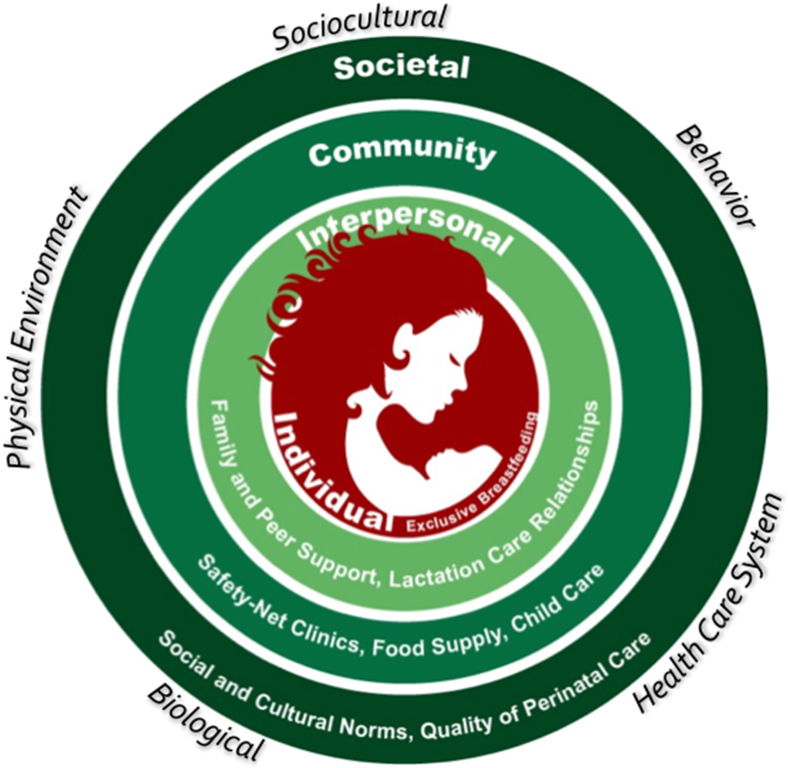
*La Cosa Más Buena* framework.

A total of 111 specific determinants were referenced across all 20 framework cells and are publicly available [27]. These specific determinants were consolidated into 53 determinant themes that were used to create the framework (Figure 1). We called it *La Cosa Más Buena* referring to “The Better Thing,” which is a play on words from the cultural norm *las dos cosas* that promotes both breastfeeding and formula feeding among Latino populations. The coalition decided to center the framework’s Individual level on the mother–infant dyad, as opposed to the mother or infant alone, because breastfeeding requires the interaction of both entities. The average number of determinant themes referenced per framework cell was 2.9 (SD = 1.7) with a range of 1–7. The most referenced level was the Individual level at 20 determinant themes, and the most referenced domain was Behavior at 14 determinant themes. The most referenced level domain cell was Individual-Behavior with 7 determinant themes, whereby the least referenced themes included the following cells with 1 each: Individual-Personal Environment, Interpersonal-Biological, Community-Sociocultural Environment, and Societal-Biological, and Societal-Behavior. Figure 2 depicts our customized graphic of the framework with the levels represented at the top of each circle, the domains revolving around the mother–infant dyad, and some specific determinants listed at the bottom of each respective circle. Determinants were mostly referenced in the working groups; however, some determinants warranted follow-up after the working group period with coalition members, the literature, and new collaborators that were engaged in the process (e.g., CEO of the Milk Bank).

### Mapping phase results

**FIGURE 3:**
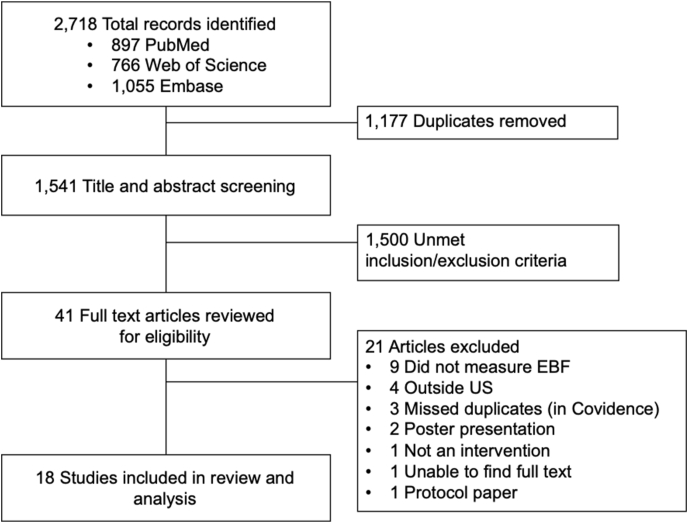
CONSORT flow diagram. EBF, exclusive breastfeeding.

The literature search through PubMed (*n =* 897), Web of Science (*n =* 766), and Embase (*n =* 1,055), yielded 2718 total potentially eligible articles (Figure 3, CONSORT diagram). After omitting 1177 duplicate articles, 1541 were included in the title and abstract screening phase. A total of 41 were selected for full-text review. After full-text reviews, an additional 21 articles were excluded because of not measuring exclusive breastfeeding, having been conducted outside the United States, manually identified duplicates, poster presentations, protocol papers, or was not an intervention. A total of 18 studies were included in the scoping review.

Study, intervention, and sample characteristics of all studies were included in the review as Supplementary Material (Supplemental Table 1). Notably, only 1 study was conducted in the Midwest United States region with the majority in the Northeast, zero of which were tested in Indiana. A balance of single-site (56% of studies) and multisite studies (44%) were conducted, mainly in the clinical setting. From almost half of studies that reported on country of origin (*n =* 9), most study samples included individuals whose country of origin was Mexico, and few enrolled individuals from Guatemala, Puerto Rico, Dominican Republic, El Salvador, Peru, Honduras, and Costa Rica in combinations. In very few studies, social factors that are increasingly being measured across health systems today (e.g., food security, financial stability, transportation status, or housing stability) were collected in studies, whereas most studies collected data on education status and marital status. Regarding intervention implementation, the most prevalent implementers were IBCLCs but only in 4 of 18 studies (22%). All other studies varied greatly in types of professions engaged as implementers (e.g., health educators, peer counselors, research staff, etc.). Most studies (*n =* 13) enrolled study samples that were entirely of Latino descent.

Three determinant themes emerged in the literature review that were otherwise not identified in our framework adaptation phase, totaling 56 determinants that created *La Cosa Más Buena* (53 from adaptation phase + 3 from mapping phase). Two of those determinants fell in the Individual-Sociocultural Environment cell (English Proficiency and Literacy; Low Socioeconomic Status), and the third emerging determinant fell in the Individual-Behavior cell (Breast Milk Handling Skills). Table 1 demonstrates the count of studies that addressed determinant themes at each cell of the framework. Most studies addressed Individual-level determinants at each domain (*n =* 11–16 studies) except for the Built Environment domain (*n =* 3 studies). At the Interpersonal level, Behavior (*n =* 11 studies) and Health Care System (*n =* 16 studies) domains were predominantly addressed. At the Community level, the Built Environment (*n =* 14 studies) and Health Care System (*n =* 15 studies) domains were addressed; and at the Societal level, most studies addressed the Health Care System domain with zero studies addressing Biological, Behavior, or Built Environment domains. Table 2 shows that extension of care, culturally relevant care, knowledge and skills, mother–infant bonding, and practitioner–dyad relationship, health care quality, and health systems design were referenced the most of all 56 determinant themes—≥13 studies each.

**TABLE 1:** Mapping determinants addressed in interventions among Latinos.

Life course domains	Levels of influence			
	Dyad	Interpersonal	Community	Societal
Biological	15 (83%)	1 (6%)	0 (0%)	0 (0%)
Behavior	16 (89%)	11 (61%)	7 (39%)	0 (0%)
Built Environment	3 (17%)	9 (50%)	14 (74%)	0 (0%)
Sociocultural Environment	11 (61%)	1 (6%)	0 (0%)	7 (39%)
Health Care System	12 (67%)	16 (89%)	15 (83%)	17 (94%)
Exclusive breastfeeding outcomes	18 (100%)	0 (0%)	0 (0%)	0 (0%)

This table lists the number of studies that addressed respective cells at least once in the study.

**TABLE 2:** Mapped determinants themes across 18 studies.

Cells (level domain)	Framework determinants	Number of studies that addressed determinants	Total references within cells across all studies
Community-Behavior	Social media influences	0	7
	Family and peer support systems	7	
Community-Biological	Obesogenic community	0	0
	Vaccination effects	0	
Community-Built Environment	Clinical environment	7	31
	Community resources	8	
	Extension of care	13	
	Resource proximity	3	
	Transportation systems	0	
Community-Health Care System	Culturally relevant care and cultural needs	14	14
	Social care and services availability	0	
Community-Sociocultural	Community norms, values, and beliefs	0	0
Individual-Behavior	Breast milk handling skills	2	40
	EBF attitudes	1	
	Infant nutrition education	11	
	Knowledge and skills	15	
	Maternal perceptions	8	
	Self-efficacy to exclusive breastfeeding	3	
Individual-Biological	Infant physiological state	0	15
	Intergenerational effects—individual	0	
	Maternal physiological state	1	
	Maternal–infant bonding	14	
	Transgenerational effects—biological	0	
Individual-Built Environment	Breastfeeding equipment	3	3
Individual-Health Care System	Health insurance	0	11
	Transportation	11	
Individual-Sociocultural	Low socioeconomic status	11	21
	English proficiency and literacy	2	
	Cultural norms, values, and beliefs	5	
	Social pressures	1	
	Transgenerational effects—sociocultural	2	
Interpersonal-Behavior	Infant feeding planning	10	15
	Intergenerational effects—interpersonal	1	
	Social support system—interpersonal	4	
Interpersonal-Biological	Intergenerational effects—interpersonal	1	1
Interpersonal-Built Environment	Household environment	8	11
	Work environment	2	
Interpersonal-Health Care System	Practitioner–dyad relationship	15	31
	Resource accessibility	5	
	Health care support systems	11	
Interpersonal-Sociocultural	Immigration effects—interpersonal	1	2
	Transgenerational effects—behavior	1	
	Transgenerational effects—sociocultural	0	
Societal-Behavior	Policies	0	0
Societal-Biological	Obesogenic society	0	0
Societal-Built Environment	Clinical structure	0	0
	Donor milk system structures	0	
	Societal structure	0	
Societal-Health Care System	Health care biases	0	44
	Health care policy	0	
	Health care quality	17	
	Health system design	17	
	IBCLC inequities	10	
Societal-Sociocultural	Social norms, values, and beliefs	2	9
	Targeted advertisement	0	
	Structural discrimination	0	
	System structures	7	

Abbreviations: EBF, exclusive breastfeeding; IBCLC, International Board Certified Lactation Consultant.

## Discussion

After successful implementation of regulations, policies, and legislation, and work-based lactation programs, suboptimal exclusive breastfeeding rates at 6 mo among Latinos in the United States persist [12,13,22,[28], [29], [30]]. The fact that we documented over 110 specific determinants across all cells of *La Cosa Más Buena* illustrates the complexity of achieving exclusive breastfeeding. Our scoping review identified key areas that could drive future innovations to increase exclusive breastfeeding among Latino populations.

As expected, many of the determinants referenced in our working groups have been described in the literature, including food insecurity, housing instability, limited transportation, social vulnerability, perceptions of milk effects, and policy support [19,[31], [32], [33], [34], [35], [36]]. Other adverse determinants spanned the broad areas of culture (e.g., *las dos cosas*), implementation (e.g., lack of bilingual workforce, distinct eligibility criteria), systems integration (e.g., low agency cross-talk, fragmented referral pathways), and human ergonomics (e.g., challenges navigating transportation systems, patient follow-through). Although these determinants are common across many health systems and communities in the United States, addressing them warrants understanding of how they operate in local contexts which vary across regions—from the populations prioritized, systems involved in implementation (e.g., health, transportation, etc.), and how people interact with those systems [37]. As an example, donor milk dispensaries installed statewide were alluded to in our working groups as potential facilitators of exclusive breastfeeding to address the common perception among Latinos that milk supply is insufficient [20,34]. This reference stimulated conversation and engagement of the local milk bank to affirm that donor milk is only used as “bridge” milk for term infants when supply is high as it is prioritized for hospitals that serve high-risk infants (e.g., preterm birth). Given that Indiana has the seventh highest infant mortality rate, using donor milk to support exclusive breastfeeding may not be a top priority, nor a scalable one. Regardless of supply, local anecdotes and the literature reflect a low affinity toward the use of donor milk among Latinos [38,39]. Nonetheless, it is the richness in the back-and-forth dialog between partners with distinct perspectives that is informative to specifications of interventions that are designed to fit the local contexts. For this reason, methods like participatory design are encouraged to engage key partners (e.g., implementers, recipients) who are most keen to everyday operations and workflows and, as a result, have rich insights into determinants that ought to be prioritized and that are also feasible within given systems [40].

As part of the scoping review to map *La Cosa Más Buena*, we found that most interventions addressed determinant themes across the Individual level or the Health Care System domain, such as mother–infant bonding, extension of care (via home visitation or telecommunication), infant nutrition education, and promoting a positive practitioner–dyad relationship—many of which are inherent in an intervention that promotes breastfeeding. As for gaps in the framework, determinants in the Biological and Sociocultural domains beyond the Individual level (e.g., obesogenic environment, community norms) were minimally addressed or not at all. Biologically, obesity is a multifactorial chronic disease that disproportionately impacts Latino populations and is associated with low breastfeeding rates [20,[41], [42], [43], [44], [45]]. Although treating obesity might result in improvements in breastfeeding outcomes, current effective treatments (bariatric surgery and antiobesity medications) are expensive, not all pregnancies are planned, and targeting obesity without targeting other determinants is likely a failed approach to achieve national breastfeeding goals (exclusivity, continuation for 2 y) [46]. Therefore, specialized interventions are needed to address adverse determinants that are unique to maternal obesity, including breastfeeding stigma, larger anatomical breast structures, latching difficulties, and delays in milk supply [20,[47], [48], [49]]. We should also note a growing field of human milk composition science, which has identified obesity- and diabetes-related biomarkers (leptin, insulin, and glucose) as predictors of breastfeeding cessation among mothers with obesity [50]. Such research will elucidate the roles of bioactive compounds that can inform future personalized approaches.

Regarding the Sociocultural domain, only 1 study addressed a related determinant across Interpersonal or Community levels. This is a missed opportunity considering the cultural norms across these levels that have been identified as strengths among Latino populations for health interventions, including *familismo* (familism), values around breastfeeding, and high intentions to breastfeed [16,51,52]. Additionally, the point of local contextualization is especially important among Latinos in the United States because of the heterogeneity in ethnic subgroups that have demonstrated differential responses to the same breastfeeding interventions (e.g., no response among Puerto Ricans compared with non-Puerto Ricans) [53]. To this end, the Midwest has been referred to as an emergent Latino community, defined as populations that settle in nontraditional immigration destinations [54,55]. As reflected in the literature and echoed by our community partners, emergent Latino communities experience unique challenges that exacerbate barriers to access to high-quality health care, including discordance in health systems (i.e., mismatch of personnel race/ethnicities or language), lack of Spanish-speaking support, or limited transportation from rural residences [54,55]. Interestingly, United States-born Latino populations demonstrate significantly lower exclusive breastfeeding rates compared with United States-born non-Hispanic White populations, which is not the case among foreign-born Latinos; to this end, higher acculturation levels have been associated with lower breastfeeding rates [36,[56], [57], [58]]. Therefore, future innovations in the United States may be enhanced by implementing transitional programs for new Latina immigrant mothers and leveraging the sociocultural values that they bring with them from other countries. Empowering and integrating immigrants as “Champions” of existing breastfeeding programs may also facilitate successful transitions through the expansion of social networks, contributing to society, and, potentially, relief from financial strain. These sociocultural determinants, alone, are complex and reinforce the need to engage key community partners in the research and intervention design processes.

Societal-level determinants influence formula use among Latino populations through its widespread availability (even as gifts upon departure of discharge in some hospitals), marketing and advertisement of human milk substitutes (sometimes targeted toward low-income groups), and social normalcy [34,35,59,60]. Policy efforts are needed to regulate and reduce the exploitative marketing of commercial milk formula [18], especially since the United States does not adhere to “the Code”—an international policy developed in 1981 by the WHO and the United Nations International Children’s Fund to regulate marketing of infant formula, other human milk substitutes, feeding bottles, and teats [21,28,61]. Other Societal-level strategies that have been associated with improved breastfeeding outcomes include the Baby-Friendly Hospital Initiative—a credentialing process for hospitals that commit to the Ten Steps for Successful Breastfeeding and comply to the Code [62]—and the infant formula shortage of 2022. Baby-Friendly initiatives in hospitals and clinics could have profound effects on exclusive breastfeeding rates at the population level but require investment from high-level executives with decision making powers as well as the capacity in personnel and system structures to maintain credentials [18,[63], [64], [65], [66]]. The recent infant formula shortage of 2022 was a natural experiment that was associated with an 11% increase in breastfeeding intentions [67]. However, this unanticipated shortage also led to increased stress and anxiety and expressions of judgment of what it means to be a good mother [68]. Nonetheless, it is clear that Societal-level changes have the potential to induce meaningful effects on exclusive breastfeeding outcomes, warranting engagement of partners that operate at this level, such as health department executives, health care CEOs, and policymakers.

Although local context specifications of interventions may not be generalizable, the processes to design, adapt, and implement interventions to address those contexts can be generalized—underscoring the importance of teams to disseminate those processes. As we used the NIMHD framework to bring together a diverse community coalition, *La Cosa Más Buena* is a tool that may be applied by others to stimulate conversations and collaborations to adapt the framework based on the local contexts of its intended use. It may also be useful as an evaluation tool, particularly at health systems that excel in achieving breastfeeding goals to reveal any leverage points that can be adopted and adapted in other systems. Future research in this field would benefit from the integration of systems thinking, human factors engineering, and dissemination and implementation science as well as reporting health outcomes in addition to breastfeeding rates [32,69,70].

### Strengths and limitations

This is the first multilevel and multidomain framework focused on exclusive breastfeeding among Latino populations. The framework adaptation process included a diverse coalition of experts in the field who serve the same local Latino communities that enabled rich discussions about interacting systems (or agencies), or systems that could interact with one another, to cater to the Latino community. It should be emphasized that *La Cosa Más Buena* is merely a list of determinants that was developed among partners in Indiana; thus, generalizability is limited but can still inform others to adopt our participatory design approaches for adapting the framework [71]. Our team included predominantly professionals in the field with 1 community consultant, and engaging community members is critical to refining the framework. We also acknowledge that not all mothers are either willing or able to breastfeed. From conversations with our community partners in addition to the literature [13,16], we appreciate the appropriateness of exclusive breastfeeding interventions among Latino populations. Additionally, scoping reviews do not entail a quality of study assessment as do systematic reviews. However, scoping reviews serve the purpose of mapping certain concepts, which was the focus of this study, and is helpful for determining whether a systematic review is warranted [24]. Last, our mapping of the framework in the scoping review was limited to the intervention as reported in respective articles.

In summary, we created a multilevel, multidomain determinants framework for exclusive breastfeeding among Latino populations. Our integrated scoping review further identified saturated areas (e.g., Individual level, Health Care System domain) and gaps in the framework (e.g., Biological and Sociocultural domains beyond the Individual level) that can inform direly needed innovations.

## Author contributions

The authors’ responsibilities were as follows – AP, RJH: conceived the study and designed the research plan; AP: held study oversight; AP, ZB, AM Miller, AA, DR, MK, AMN, AML, KH, TP, AS, CS, FB, JAP, IC, KB, KS, AM Mays, SL, CL, RD, EL, FK, EMC, KC, AM: conducted the research; AP, ZB, AMM, AA: analyzed the data; AP: wrote the first draft of the article; and all authors: read, edited, and approved the final manuscript.

## Data availability

Data are publicly available at https://osf.io/e635z/.

## Funding

The authors reported no funding received for this study.

## Conflict of interest

The authors report no conflicts of interest.
