# Supplementary material for: Co-creating and Mapping an Exclusive Breastfeeding Framework among Latino Populations in the United States: An Integrated Framework Adaptation Process and Scoping Review
Source: Adv Nutr. 2025 Jul 19;16(9):100483. doi: 10.1016/j.advnut.2025.100483 (PMC12358663; doi:10.1016/j.advnut.2025.100483)
Supplement: Multimedia component 1 [file mmc1.docx]

**Title**: Co-creating and mapping an exclusive breastfeeding framework among Latino populations in the US: An integrated framework adaptation process and scoping review

**First Author**: Armando Peña

| **Table S1**. Sample and intervention characteristics of 18 studies in scoping review | | | |
| --- | --- | --- | --- |
| Level of Study | Study Characteristics | Number of total studies, n (%) | Percent of total studies |
| Study | Study type |  |  |
|  | *Randomized trial* | 14 | 78% |
|  | *Non-randomized trials* | 4 | 22% |
|  | Perinatal phase study start |  |  |
|  | *Prenatal* | 15 | 83% |
|  | *Postnatal* | 3 | 17% |
|  | Powered to detect significant differences in EBF |  |  |
|  | *Yes* | 4 | 22% |
|  | *No* | 12 | 67% |
|  | *Unknown* | 2 | 11% |
|  | US state of study |  |  |
|  | *California* | 2 | 11% |
|  | *Colorado* | 1 | 6% |
|  | *Connecticut* | 3 | 17% |
|  | *Kansas* | 1 | 6% |
|  | *Kentucky* | 1 | 6% |
|  | *New Jersey* | 1 | 6% |
|  | *New York* | 3 | 17% |
|  | *Pennsylvania* | 1 | 6% |
|  | *Tennessee* | 1 | 6% |
|  | *Texas* | 2 | 11% |
|  | *Virginia* | 2 | 11% |
|  | US region |  |  |
|  | *Northeast* | 8 | 44% |
|  | *Midwest* | 1 | 6% |
|  | *West* | 3 | 17% |
|  | *Southeast* | 4 | 22% |
|  | *Southwest* | 2 | 11% |
| Sample | Total participants randomizedª, mean N (N range) | 250 (36 - 533) | |
|  | Maternal age^a,b^, Mean | 26 y | |
|  | Proportion of Latinos in participant samples |  |  |
|  | *100%* | 13 | 72% |
|  | *90-99%* | 0 | 0% |
|  | *80-89%* | 2 | 11% |
|  | *70-79%* | 2 | 11% |
|  | *60-69%* | 0 | 0% |
|  | *>50-59%* | 1 | 6% |
|  | Country of origin in participant samples |  |  |
|  | *Costa Rica* | 1 | 6% |
|  | *Dominican Republic* | 1 | 6% |
|  | *Guatemala* | 2 | 11% |
|  | *Honduras* | 2 | 11% |
|  | *El Salvador* | 2 | 11% |
|  | *Mexico* | 7 | 39% |
|  | *Peru* | 1 | 6% |
|  | *Puerto Rico* | 1 | 6% |
|  | *Not known* | 9 | 50% |
|  | *Reported sociodemographic study characteristics* |  |  |
|  | *Education* | 12 | 67% |
|  | *Marital status* | 10 | 56% |
|  | *Employment status* | 6 | 33% |
|  | *Household income* | 4 | 22% |
|  | *Household members* | 3 | 17% |
|  | *WIC participation* | 3 | 17% |
|  | *Household food insecurity* | 2 | 11% |
|  | *Insurance* | 2 | 11% |
|  | *Prior breastfeeding experience* | 2 | 11% |
|  | *Acculturation to US* | 1 | 6% |
|  | *Native language* | 1 | 6% |
|  | *Perceived breastfeeding support* | 1 | 6% |
|  | *Planning to return to work* | 1 | 6% |
|  | *SNAP participation* | 1 | 6% |
|  | *US born* | 1 | 6% |
|  | *Vocational training* | 1 | 6% |
| Intervention | Number of sites |  |  |
|  | *Single site* | 10 | 56% |
|  | *Multi-site* | 8 | 44% |
|  | Setting |  |  |
|  | *Clinical* | 10 | 56% |
|  | *Home-based* | 2 | 11% |
|  | *Clinical and home-based* | 3 | 17% |
|  | *Community organziation and home-based* | 3 | 17% |
|  | Mode of Intervention Delivery |  |  |
|  | *In person* | 7 | 39% |
|  | *Hybrid (In person and phone)* | 8 | 44% |
|  | *Phone* | 3 | 17% |
|  | Intervention perinatal periods |  |  |
|  | *Prenatal* | 1 | 6% |
|  | *Postnatal* | 4 | 22% |
|  | *Prenatal and postnatal* | 13 | 72% |
|  | *Theoretical frameworks or behavioral theories used* |  |  |
|  | *Social Cognitive Theory* | 2 | 11% |
|  | *Health Belief Model* | 2 | 11% |
|  | *Health Promotion Model* | 1 | 6% |
|  | *Theory of Community Empowerment* | 1 | 6% |
|  | *Did not report or unknown* | 14 | 78% |
|  | Mode of EBF Measurement |  |  |
|  | *Questionnaire/Survey* | 16 | 89% |
|  | *Interviews* | 2 | 11% |
|  |  |  |  |
|  | Intervention Implementers |  |  |
|  | *Lactation consultant (IBCLC)* | 4 | 22% |
|  | *Health educators* | 2 | 11% |
|  | *Peer counselors* | 2 | 11% |
|  | *Research staff* | 2 | 11% |
|  | *Registered nurse* | 2 | 11% |
|  | *Peer educators* | 1 | 6% |
|  | *Principal investigator* | 1 | 6% |
|  | *Promotora* | 1 | 6% |
|  | *Registered dietitian* | 1 | 6% |
|  | *Family support worker* | 1 | 6% |
|  | *Community health worker* | 1 | 6% |
|  | *Maternal-infant health outreach worker* | 1 | 6% |
|  | *Undergraduate student* | 1 | 6% |
| Outcomes | Significant improvements in EBF rates^c^ |  |  |
|  | *Yes* | 8 | 44% |
|  | *No* | 10 | 56% |
| ^a^Of the studies that were randomized trials ^b^Of the studies that reported maternal age ^c^As reported by criteria used in study  US: United States, EBF: exclusive breastfeeding, WIC: Supplemental Nutrition Assistance Program for Women, Infants, and Children, SNAP: Supplemental Nutrition Assistance Program, IBCLC: International Board Certified Lactation Consultant | | | |
